# Avoidable hospitalizations in Brazil and Portugal: Identifying and comparing critical areas through spatial analysis

**DOI:** 10.1371/journal.pone.0219262

**Published:** 2019-07-12

**Authors:** João Victor Muniz Rocha, Carla Nunes, Rui Santana

**Affiliations:** 1 Escola Nacional de Saúde Pública, Universidade NOVA de Lisboa, Lisbon, Portugal; 2 Centro de Investigação em Saúde Pública, Universidade NOVA de Lisboa, Lisbon, Portugal; The University of the South Pacific, FIJI

## Abstract

**Background:**

Hospitalizations for ambulatory care sensitive conditions have been used to assess the performance of primary health care. Few studies have compared geographic variation in rates of avoidable hospitalizations and characteristics of high-risk areas within and between countries. The aim of this study was to identify and compare critical areas of avoidable hospitalizations in Brazil and Portugal, because these countries have reformed their primary health care systems in recent years and have similar organizational characteristics.

**Methods:**

An ecological study on hospitalizations for ambulatory care sensitive conditions produced in Brazil and Portugal in 2015 was used. Geographic variation of rates were analyzed and compared at the municipal level. A spatial scan statistic was employed to identify clusters with higher risk of hospitalizations for acute and chronic conditions in each country separately. Socioeconomic and primary health care characteristics of critical areas were compared to non-critical areas.

**Results:**

There were high variations in rates of avoidable hospitalizations within and between Brazil and Portugal, with higher variations found in Brazil. A more evident pattern of rates was found in Portugal. Rates and cluster distribution of acute and chronic conditions had significant agreement for both countries. The differences in primary health care and socioeconomic characteristics between areas identified as high risk clusters and non-clusters varied between category of conditions and between countries.

**Conclusion:**

Brazil and Portugal presented expressive regional differences with respect to rates of avoidable hospitalizations, indicating that there is room to improve by reducing such events in both countries. Different areas presented distinct interactions between primary health care, socioeconomic characteristics, and avoidable hospitalizations. Results indicate that the primary health care reforms, with similar organizational characteristics in different contexts, did not produce similar results either between or within countries. Possible actions to reduce these events should be defined at a local level.

## Introduction

Ambulatory care sensitive conditions (ACSC) are conditions for which timely and effective care in the ambulatory setting could potentially avoid the need for hospitalization. For this reason, hospitalizations due to ACSC have been extensively analyzed in health care research, and their usefulness has been endorsed by national and international organizations. This indicator can also be used by health managers to assess performance of the primary health care (PHC) delivery system within the broader health system [1–5].

The interaction of different dimensions of the health system and how they produce outcomes is the basis for the analysis of ACSC. The inputs for health assessment are related to the design, organization and management of health systems. Such inputs lead to performance outcomes related to access, quality, coordination and efficiency of health system delivery [5,6]. These outcomes lead to impacts in health, namely the avoidable morbidity represented by hospitalizations for ACSC. When measuring the performance of health services delivery through avoidable hospital admissions, it is important to note the way elements of the social, economic, political and geographic dimensions interact with individual biological factors and behaviors, shaping health status.

Detection of geographical areas which present higher rates of hospitalization for ACSC can identify critical areas which should be focused on—e.g., health managers should conduct deeper epidemiological investigations and health policy interventions [7,8]—because it is expected that there are inequities in distribution and access to health care and a low capacity of PHC for preventing, diagnosing, treating, and managing these conditions [4,5,9].

Wide geographic variations in rates of hospitalizations for ACSC were found in Italy [9], London [10], Madrid [11] and Switzerland [12], despite the existence of universal health care systems. In France, Germany and Italy, different geographic patterns between acute and chronic ACSC were also found [9,13,14]. Acute and chronic conditions have distinct levels of prevention, management and treatment [5,15]; while acute conditions could be avoided by early diagnosis and treatment, the management of chronic conditions can depend on referral to a specialist and an appropriate follow-up [14,16]. Chronic conditions can be the result of long periods of some specific health behaviors or a gradual deterioration of the patient’s condition, indicating that there are different degrees of preventability among commonly considered ACSC.

Previous evidence indicates that geographic variation in avoidable hospitalization rates is associated with both lower physician supply and PHC center availability in areas with higher risk [8,12,14]. In addition, socioeconomic and health characteristics of the population (such as rurality, education, and economic level) also play an important role in geographic variations in the rates of these hospitalizations [8,10,12,17]. Comparing characteristics of critical areas can help us understand variables associated with a higher risk of avoidable hospitalization [18].

Only a few studies have analyzed variations in rates of hospitalizations for ACSC and associated factors between countries, taking into consideration their health care systems; these have mostly focuses on developed countries. A study of five European countries (Denmark, England, Portugal, Slovenia and Spain) found substantial variation between and within countries. The findings indicated that there was a significant association between the proportion of people with low levels of education and higher rates of avoidable hospital admissions for Denmark, Portugal, Slovenia and Spain [19]. Another comparative study analyzed hospitalizations for ACSC in Italy and Germany, because these countries have sociodemographic and economic similarities, but have different models of organization of their health care systems [14]. The study found clear patterns of higher rates of hospitalization for chronic ACSC in specific regions of both countries; those regions have a lower GDP per person and lower levels of healthcare facility resources. Less clear patterns and not statistically significant correlations were found for acute ACSC.

Different countries have carried out reforms of their health care systems, in the interests of improving the quality and efficiency of care. Brazil and Portugal have reformed their PHC in recent years to improve accessibility, efficiency, and quality of health care, both using a similar approach based on family health units (FHUs), in which multidisciplinary teams provide community-based care, with a payment system that rewards performance [20,21].

These reforms were adopted following the positive results of innovative experimental projects on PHC services adopted in Brazil and Portugal, given the health needs of the population. These experiences were mostly based on the autonomy of FHU teams, the close contact with the community and pay for performance schemes. Brazil and Portugal also have coverage differential across the countries: in Portugal the existing FHUs are concentrated along the coastal area, which is more densely populated [21,22]; in Brazil there are difficulties in promoting access to and consolidating a proactive model care of primary health care in large urban centers [23]. There are also difficulties related to insufficiency and unequal distribution of human resources, which can be partially explained by inequities in socioeconomic contexts (such as the knowledge of health management and of the organization of the health system), choice of health providers and human resources distribution [22,24].

In both countries, the FHUs coexist with traditional PHC units, mainly characterized in Brazil by services provided in response to spontaneous demand based on physician-centered care and, in Portugal, by the lack of incentive mechanisms and autonomy for health teams [25,26]. Both countries have universal health systems with decentralized organization, indicating that management of the PHCs happens at the regional level [27,28]. On the other hand, both countries have considerable differences in their level of development, population compositions according to age group [29], life expectancies, causes of years-of-life-lost [30,31], economic inequality, poverty rates [29,32] and educational levels [33].

Table 1 presents selected primary health care and socioeconomic characteristics of Brazil and Portugal. Information on coverage of FHU and physician supply are from the Brazilian Health System Informatics Department, Biscaia and Heleno (2017) [34] and the Portuguese Central Administration of the Health System [35]. Information on proportion of elderly, life expectancy at birth, GDP per capita and Gini index are from the World Bank open data. Information on level of education comes from the OECD [33] and from rurality comes from the Brazilian Institute of Geography and Statistics and the Statistics Portugal database.

**Table 1:** Sample characteristics.

	Brazil	Portugal
**Primary Health Care**		
Objective of PHC reform	Reorient the work process in primary health care, articulated to the family and community context, to increase the resolution and impact on the health situation of the population.	Improve primary health care accessibility, efficiency, quality and continuity of care and increase the satisfaction of professionals and citizens.
Coverage of FHU	Family health teams: 39,675 Population covered: 124,126,038 (60.7%) (2015)	Family health units: 459 Population covered: 5,361,959 (54.5%) (2016)
Physician supply *Primary care physicians per 1*,*000 people*	0.36 (2015)	0.66 (2015)
**Socioeconomic characteristics**		
Proportion of elderly *Proportion of people aged 65 years or older*	8.0% (2015)	21.1% (2015)
Life expectancy at birth	75 years (2015)	81 years (2015)
Rurality *Proportion of population living in rural areas*	19.5% (2015)	12.7% (2015)
GDP per capita *In US$ Purchase Power Parity (PPP)*	US$ PPP 15,656 (2015)	US$ PPP 29,523 (2015)
Gini índex	51.3 (2015)	35.5 (2015)
Level of education *Proportion of population aged 25–64 years with primary education or below*	37% (2015)	32% (2016)

Both countries have sufficient similarities in objectives, organization and coverage of primary health care services, and differences in socioeconomic characteristics means within and between countries, to make the comparison of geographic dynamics of hospitalizations for ACSC suitable, opportune, and relevant. Other countries might face similar health system challenges and the comparative approach can provide information on the potential to resolve difficult health care delivery problems. To identify and characterize critical areas of avoidable hospitalizations is a first step to later target those and reduce the overall burden of ACSC. As the two countries have similar PHC organization, this analysis can provide hints on what dimensions in PHC supply and socioeconomic characteristics should be the focus of subsequent targeted actions. The objective of this study was to identify critical areas of avoidable hospitalizations in Brazil and Portugal in 2015, considering both acute and chronic ACSC. A secondary goal was to characterize and compare these areas with non-critical areas, considering socioeconomic and health services characteristics.

## Materials and methods

### Study design and data sources

This is an ecological cross-sectional study on hospitalizations for ACSC occurring in adult populations in Brazil and Portugal in 2015. The unit of analysis in this study is the municipality: 5,570 for Brazil and 278 for mainland Portugal. The average size of the municipal units in Brazil is 1,526 km^2^, and the average population was 36,706 (minimum: 813; maximum: 11,967,824; SD: 215,590). The average size of the municipal units in Portugal is 320 km^2^, and the average population was 35,393 (minimum: 1,717; maximum: 504,471; SD: 56,807).

This study used the hospitalization databases provided by the Brazilian Hospital Admissions Information System and the Portuguese Central Administration of the Health System for the year 2015. A total of 11,522,004 and 1,000,670 hospitalizations were registered for Brazil and continental Portugal in 2015, respectively. Both databases are produced to reimburse hospitals and, therefore only cover public hospitals. In both countries, the physicians evaluate the patients and determine the principal and secondary diagnosis code, according to the International Classification of Diseases (ICD) (the 9^th^ revision for Portugal and the 10^th^ revision for Brazil). In addition, external auditors frequently check the hospital data bases, to ensure quality and identify potential errors. The data is anonymized and was analyzed according to the municipality of residence of the patient.

Data on PHC supply and the socioeconomic characteristics of municipalities were selected according to the literature and data availability, and the sources were the Brazilian Institute of Geography and Statistics (IBGE), the Brazilian Health System Informatics Department (DATASUS), the Statistics Portugal database (SP), and the Portuguese Central Administration of the Health System (ACSS). Table 2 details the variables used and data sources. The ecological variables were: proportion of people aged 65 years or older in the population, population density, proportion of people living in rural areas, economic level (mean of household income in Brazilian reais for Brazil; relative purchase power with the national purchase power used as reference (= 100) for Portugal), proportion of people with low education, physician supply in FHUs and in PHC centers in general, and population coverage of FHUs (for Brazil, this was the number of family health teams * 3,450/population and, for Portugal, this was the number of users registered at FHUs/population). Primary Health Care data for Portugal was retrieved from the periodic publication on number of patients registered on PHC services [35].

**Table 2:** Variables information.

Variable	Description	Brazil		Portugal	
		Source	Year	Source	Year
Primary health care reform quantitative characteristics					
Physician supply in FHU	Proportion of physicians in FHU per 1,000 population	DATASUS	2015	ACSS	2015
Physician supply in PHC	Proportion of physicians in PHC per 1,000 population	DATASUS	2015	ACSS	2015
FHU coverage	(Number of Family Health Teams X 3,450)/Population (%) (for Brazil) Number of users registered on FHU/Population (%) (for Portugal)	DATASUS	2015	ACSS	2015
Socioeconomic characteristics					
Proportion of elderly	Proportion of people aged 65 years or older (%)	IBGE	2015	SP	2015
Population density	Number of habitants per km^2^	IBGE	2015	SP	2015
Rurality	Proportion of people living in rural areas (%)	IBGE	2010	SP	2011
Economic level	Mean of household income (for Brazil) Relative Purchase power, with the national used as reference (= 100) (for Portugal)	IBGE	2010	SP	2015
Education level	Proportion of people with no education or incomplete 1^st^ grade level (%) (for Brazil) Proportion of people with no education (%) (for Portugal)	IBGE	2010	SP	2011

### Definition of hospitalizations for ACSC

The definition of which hospitalizations were avoidable was determined according to the methodology of the US Agency for Healthcare Research and Quality (AHRQ), which identifies prevention quality indicators (PQIs) according to the codes of the principal and secondary diagnoses (AHRQ). This methodology was applied for all admissions of patients aged 18 years and older; it excluded obstetric admissions and transfers from other health care facilities. Cases with missing values for the variables age, sex, diagnosis, and municipality of residency were also excluded. This list has a solid theoretical basis, is periodically revised for inclusion and exclusion of cases, and can be applied for both ICD-9 and ICD-10. The use of a single list allows for comparison between both countries.

Analysis was performed separately for the composite indicators PQI 91 (acute conditions) and PQI 92 (chronic conditions). The acute conditions analyzed by this methodology were bacterial pneumonia, urinary tract infection, and dehydration. The chronic conditions were hypertension, congestive heart failure, chronic obstructive pulmonary disease (COPD) or asthma in older adults, asthma in younger adults, short-term and long-term complications of diabetes, uncontrolled diabetes, and lower-extremity amputation among diabetics. Details on disease codes used and methods of calculation can be found in the AHRQ guidelines [3].

### Spatial statistical analysis

Rates of hospitalizations for ACSC were presented as number of hospital admissions per 100,000 people over 18 years, as defined by the AHRQ methodology. Descriptive statistics, percentiles, coefficient of variation, and ratio of variation were used to visualize rates and geographic variation of ACSC rates across Brazil and Portugal for each category of ACSC. Spearman’s correlation was used to assess the relationship between rates of acute and chronic ACSC in both countries.

A spatial scan statistic was employed to identify clusters with higher risk of hospitalizations for acute and chronic ACSC in each country separately. The spatial scan statistic employed is a methodology proposed by Kulldorff [36] to test if the number of cases were randomly distributed across different circular windows or if significant spatial clusters exists, according to the corresponding relative risk (RR). The Poisson model was employed as it deals with a discrete variable (number of hospitalizations). The spatial scan statistic is based on a maximum likelihood ratio for each potential cluster, to test the hypothesis of clustering against the hypothesis of uniformity. One important assumption was the scan through circular window shapes, as there is no evidence of the presence of other specific shapes (default). The maximum spatial cluster size was defined as 20% of the population at risk; this parameter identifies clusters in useful sizes for the development of local strategies. The likelihood p-value for the hypothesis test was estimated using Monte Carlo simulations (999 simulations), as the exact distribution of the test statistic cannot be defined. Kulldorff [36] provides more details on the statistical procedure.

A chi-square test was used to analyze if there was a relationship between clusters of acute and chronic ACSC in each country.

The non-parametric Mann-Whitney U-test was performed to compare if significant differences for the socioeconomic variables and regional PHC quantitative measures existed between areas identified as clusters with high-risk of hospitalization for ACSC and non-cluster areas, for each category of ACSC. The spatial scan analysis was performed using SatScan 9.4 and statistical analysis was carried out using IBM SPSS 21.0.

## Results

An overview of hospitalization for ACSC in Brazil and Portugal is presented in Table 3. A total of 836,837 and 99,417 million avoidable hospital admissions were registered in Brazil and Portugal, respectively. The distribution of those hospitalizations according to the category of condition was similar in both countries (59.9% and 56.6% of the hospitalizations for ACSC were due to acute conditions in Brazil and Portugal, respectively). Although Portugal presented higher rates of hospitalizations of ACSC, Brazil presented higher coefficients and ratios of variation for both categories of conditions, indicating more heterogeneity in the distribution of rates among municipalities in that country. In Brazil, the highest variation was for chronic conditions, while in Portugal it was for acute. The Spearman correlation between rates of acute and chronic ACSC across municipalities showed a positive association for both countries, indicating agreement between rates for both categories of ACSC.

**Table 3:** Rates and variation of hospitalizations for ACSC, by category and country, 2015.

		Brazil		Portugal	
		Acute	Chronic	Acute	Chronic
N	Adult Population	139,901,201		7,928,764	
	Total hospitalization cases	11,522,004		1,000,670	
	Total hospitalization rate (per 100,000 adults)	8,235.81		12,620.76	
	Hospitalizations for ACSC	836,837		99,417	
	(% of all hospitalizations)	(7.26%)		(9.94%)	
	Per category	501,377	335,460	56,245	43,172
	(% of all hospitalizations for ACSC)	(59.9%)	(40.1%)	(56.6%)	(43.4%)
Rates	Rate per 100,000 adults	358.38	239.78	709.38	544.50
	Minimum	0. 00	0.00	212.77	221.02
	Percentile 5	58.33	36.95	351.19	335.38
	Percentile 25	189.46	120.77	596.13	452.32
	Percentile 50	371.89	243.74	792.43	566.70
	Percentile 75	688.66	495.31	1,082.36	724.35
	Percentile 95	1,530.01	1,330.85	1,639.41	1,032.15
	Maximum	7,662.79	6,589.39	3,573.84	1,742.46
Variation	Coefficient of Variation	0.98	1.23	0.47	0.39
	Ratio Max/Min			16.80	7.89
	Ratio P95/P5	26.24	36.03	4.67	3.08
	Ratio P75/P25	3.64	4.11	1.82	1.61
Correlation	Spearman’s coefficient (ρ)	0.562 (p < 0.001)		0.536 (p < 0.001)	

**Fig 1:**
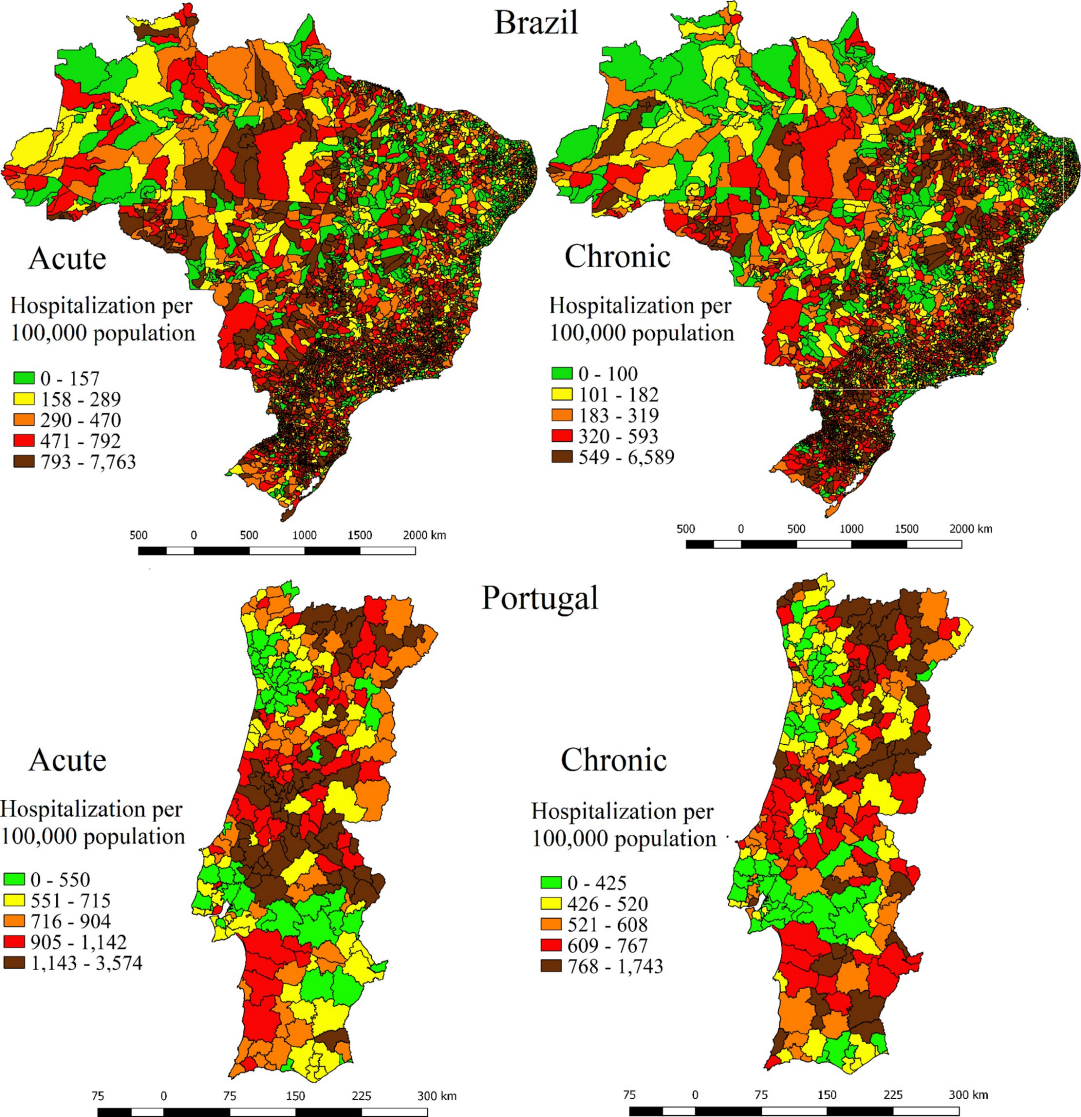
Distribution of ACSC hospitalizations rates by quintiles in Brazil and Portugal, 2015.

Fig 1 presents the geographical distribution of ACSC hospitalization rates in quintiles for Brazil and Portugal, respectively. In Brazil, municipalities in the northeast region had lower rates of acute ACSC hospitalizations. There was a concentration of municipalities with higher rates of acute ACSC hospitalizations in the center of the south half of the country, as well as in the middle of the northern region. Conversely, municipalities in the northern region had lower rates of chronic admissions. The coastal municipalities of Brazil had lower rates of avoidable hospitalizations for both acute and chronic ACSC.

In Portugal, municipalities close to Lisbon had lower rates of hospitalizations for both acute and chronic ACSC; however, the city of Lisbon itself was an exception, with higher rates for both categories. For hospital admissions due to acute conditions, the north half of the country comprised most of the municipalities with higher rates, especially in the center region. For chronic ACSC, the north–south pattern was not as evident, because municipalities in the southern region presented higher rates. Municipalities in the northern half of the country presented higher rates of both categories of conditions, especially in municipalities close to the border with Spain.

**Fig 2:**
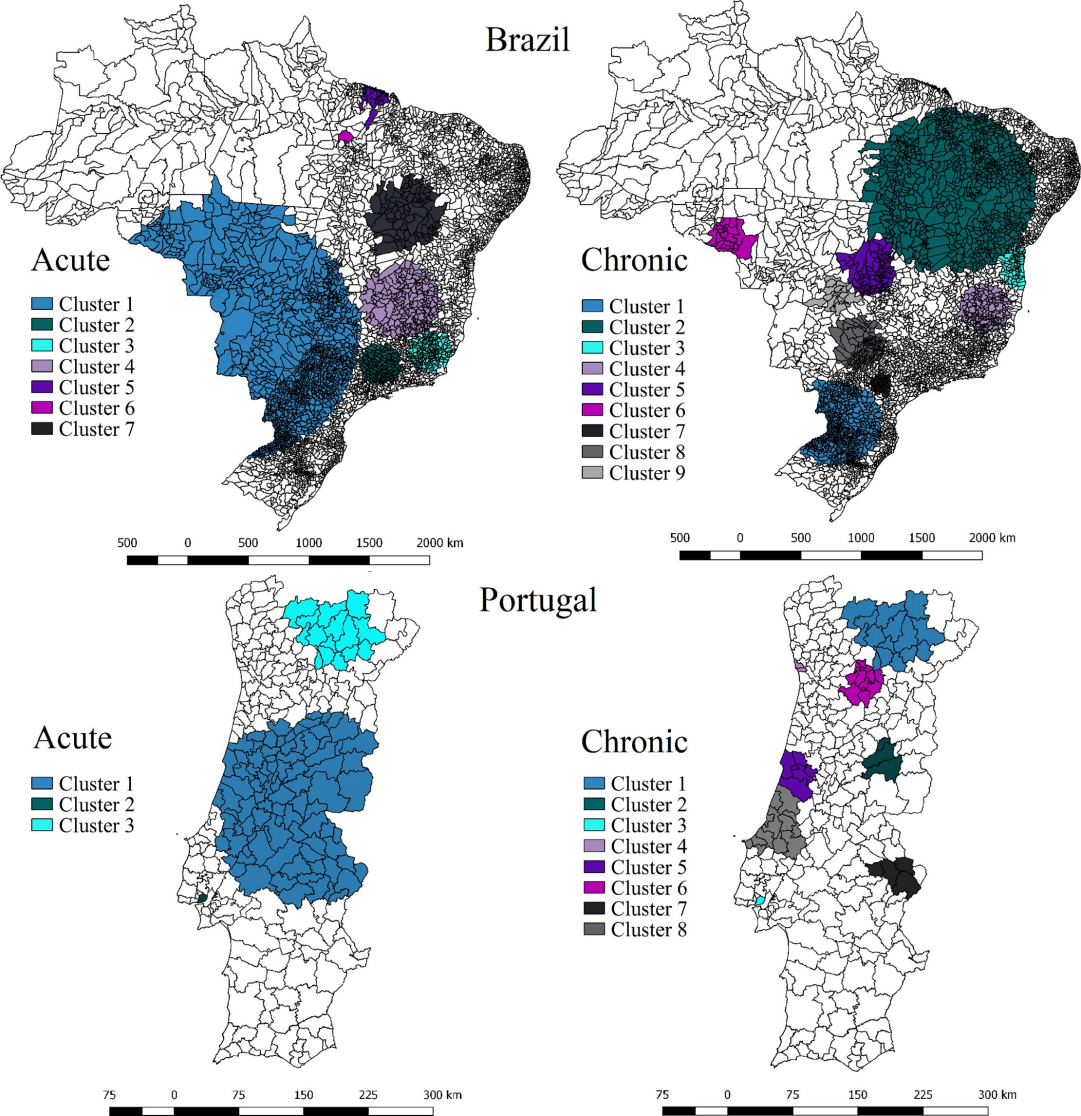
Distribution of clusters of high risk of ACSC hospitalizations in Brazil and Portugal, 2015.

Fig 2 indicates where clusters of high risk of avoidable hospitalizations were located in Brazil and Portugal. The chi-square test indicated that there was an agreement between municipalities constituting clusters of acute and chronic ACSC for Brazil (χ^2^ = 39.801, p<0.001) and Portugal (χ^2^ = 18.436, p<0.001).

In Brazil, seven clusters were identified as having high risk of hospitalization for acute ACSC. The biggest cluster comprised 1,413 municipalities, covering the center region of the country (RR = 1.83). Four other clusters were located in the interior of the southeast and northeast regions. Nine clusters with high risk for chronic ACSC where identified; the biggest one had 669 municipalities and was located in the interior of the northeast region (RR = 2.67). The other clusters were located in the interior of the southern half. There were 1,021 municipalities that were part of both acute and chronic clusters.

In Portugal, three clusters of high risk of hospitalization for acute ACSC were identified; the biggest one was in the center of the country (RR = 1.76) and the second biggest one comprised 15 municipalities of the northern region (RR = 1.82); these municipalities also composed the biggest cluster for chronic ACSC (RR = 2.04). Of the nine clusters identified for chronic ACSC, most of these were located in the central and northern regions. The spatial scan test identified Lisbon as a cluster with high risk for both categories of ACSC. There were 35 municipalities that were part of both acute and chronic clusters.

Table 4 presents the means and standard deviation for measures of socioeconomic and PHC supply characteristics of municipalities; the Mann-Whitney U-test was used to indicate if the difference in the quantitative values of the ecological variables between cluster and non-cluster was significant.

**Table 4:** Comparison of ecologic variables means between high risk clusters and no clusters using the Mann-Whitney U-test, by country and category, 2015.

	Brazil				Portugal			
Ecologic variables	Acute ACSC		Chronic ACSC		Acute ACSC		Chronic ACSC	
Mean (standard deviation)	High Risk Cluster N = 2,239	Non-cluster N = 3,331	High Risk Cluster N = 2,258	Non-cluster N = 3,312	High Risk Cluster N = 109	Non-cluster N = 169	High Risk Cluster N = 54	Non-cluster N = 224
Physician supply in FHU	**0.28** **(0.23)**	**0.32** * (0.22)	**0.32** **(0.24)**	**0.29** * (0.21)	**0.16** **(0.27)**	**0.27** * (0.29)	0.19 (0.27)	0.24 (0.29)
Physician supply in PHC	0.51 (0.43)	0.47 (0.37)	0.48 (0.37)	0.5 (0.41)	0.74 (0.19)	0.68 (0.19)	0.72 (0.16)	0.7 (0.19)
FHU coverage	87.13 (22.72)	86.77 (23.9)	**92.04** **(17.8)**	**83.42** * (26.03)	**21.6** **(36.05)**	**36.65** * (38.27)	25.4 (36.72)	32.04 (38.35)
Proportion of elderly	**13.94** **(3.4)**	**12.72** * (3.66)	**14.02** **(3.58)**	**12.66** * (3.53)	**27.91** **(5.16)**	**22.55** * (5.57)	25.7 (4.5)	24.4 (6.3)
Population density	**39.95** **(94.69)**	**167.11** * (777.53)	**27.27** **(41.32)**	**176.48** * (781.3)	**117.84** **(499.86)**	**423.08** * (974.49)	317.3 (1019.2)	300.05 (785.6)
Rurality	**52.42** **(35.67)**	**54.60** * (32.49)	**61.70** **(33.02)**	**48.29** * (33.28)	**41.51** **(20.71)**	**25.64** * (24.09)	38.83 (23.16)	30.18 (24.03)
Economic level	**556.43** **(182.48)**	**434.28** * (258.96)	**465.81** **(234.18)**	**495.41** * (241.33)	77.84 (18.28)	82.78 (18.86)	80.01 (26.28)	81.04 (16.52)
Education level	**35.44** **(8.98)**	**39.93** * (12.59)	**40.06** **(10.93)**	**36.81** * (11.67)	**16.83** **(4.73)**	**13.72** * (5.06)	15.97 (4.76)	14.69 (5.23)

* Significant difference by Mann-Whitney U-test between means of non-cluster when compared to high-risk cluster (p<0.001)

In Brazil, the mean proportion of elderly was greater for municipalities belonging to clusters with high risk of hospitalization for acute and chronic ACSC than for non-cluster municipalities. For the variables rurality, economic level, and education level, the differences between cluster and non-cluster municipalities were the opposite for acute and chronic ACSC. The Mann-Whitney test indicates that there were no differences in physician supply in PHC in general between cluster and non-cluster municipalities, but the difference in physician supply in FHUs was significant and opposite between both categories of ACSC.

For Portugal, the results indicate that there were no differences between chronic ACSC clusters and non-cluster municipalities for any of the parameters. For acute ACSC, municipalities belonging to a high risk cluster had a greater mean proportion of elderly, people living in rural areas, and people with low education level. The mean proportion of physician supply in FHUs and coverage of FHUs was lower for high risk municipalities than in non-cluster municipalities. For both Brazil and Portugal, the population density was significantly lower for high risk cluster municipalities for both categories.

## Discussion

### Key findings

The results of this study show that: (i) there are high variations in rates of hospitalizations for ACSC within and between Brazil and Portugal, with higher variations found in Brazil; (ii) there is a more evident pattern of rates in Portugal (with the northern half of the country presenting higher rates); there is no clear pattern in Brazil, only that the northern region had fewer municipalities identified as high risk clusters; (iii) the differences in PHC supply and socioeconomic characteristics between areas identified as high risk clusters and the rest of each country varied between category of ACSC and between Brazil and Portugal; and (iv) rates and cluster distribution of acute and chronic ACSC had a significant agreement between them for both countries. The results presented here agree with previous studies that indicate that hospitalizations for ACSC vary across geographic units and have different associated factors [8,12–14,19].

Regional variations in distribution of hospitalizations for ACSC, both within and between Brazil and Portugal, indicate a possible difference in the underlying factors associated with avoidable hospitalizations and, consequently, which interventions could be more successful for reducing such admissions. Given the use of hospitalizations for ACSC as a performance indicator, it is expected that the variations between and within countries indicate differences in the accessibility and quality of PHC service delivery. Despite a similar approach to providing PHC and similar ACSC hospitalization composition, Brazil and Portugal have very distinct dynamics with regard to mean values of PHC supply and coverage between critical and non-critical areas. In both countries, areas identified as clusters at high risk of acute ACSC had a lower supply of physicians in FHUs, but for chronic conditions these areas had a higher supply in Brazil and no difference for Portugal.

Some studies in Brazil have found an association between the expansion of FHUs and lower ACSC hospitalization rates (even when controlled for socioeconomic factors) [37–39]. Conflicting results on the association of the impact of FHUs on ACSC were found for different regions of Brazil [40], corroborating the idea of variability of ACSC and associated factors across the country. It is important to emphasize that the choice of methodology used to select ACSC codes leads to differences in the results [41,42]. Previous studies in Brazil used the country-specific list developed in 2009, which includes conditions not considered in this study, such as vaccine-preventable conditions, angina, gastroenteritis, nutritional deficiencies, and cellulitis, among several others [43].

In Portugal, high-risk clusters for acute ACSC had lower coverage of FHUs and lower physician supply compared with non-cluster areas, indicating that the FHUs might be associated with lower rates of avoidable hospitalizations for acute conditions. This difference however, could be due to other unobserved factors that are associated with where the FHUs were implemented. Although the supply of primary care physicians is a notable component of access [44], similarities and differences in other dimensions of PHC between countries and for smaller geographic regions should be explored in future studies.

Previous studies have found that the geographic variation in avoidable hospital admission rates were more associated with socioeconomic and health characteristics of the population than with quantitative measures of PHC supply [10,17]. For Brazil and Portugal, there were significant differences in the mean values of both PHC supply and socioeconomic characteristic variables between critical and non-critical areas. These differences indicate the existence of complex dynamics leading to the variation in rates and existence of critical areas. This complexity makes the cross-country learning more difficult and it impacts the interpretation of ACSC as an indicator for performance assessment.

In Brazil, municipalities belonging to high-risk clusters of acute avoidable hospitalizations presented higher economic levels and education levels than non-cluster municipalities. At first glance, such direction of association seems contrary to what is expected and discussed in the literature [45–47]. However, some studies have found that higher economic and education levels in Brazil are associated with higher rates of hospitalizations in general [48,49]. These studies suggest that, in Brazil, people with higher economic levels have better access to health services, including hospitalizations, either because of their understanding of the health system or their financial situation. Therefore, hospitals are used as the preferential access point to the health system for this socioeconomic group.

In Portugal, municipalities in critical areas for acute avoidable hospitalizations presented lower education levels than non-critical municipalities. Low education may lead to decreased quality of life (due to difficulties in obtaining well-paid employment and accessing goods and services), and can hinder the capacity to manage one’s own health and adopt healthy lifestyle and behaviors [50]. While the effect of education on ACSC hospitalizations in Portugal was the same as found in previous studies [46,51], in Brazil the inverse was found for acute conditions. Whether this is a reflection on PHC and hospital use or associated with other health determinants or health behavior should be explored further.

Municipalities in critical areas had a higher proportion of elderly and lower population density mean in both countries. The former reflects a concerning situation given the ageing of the population globally, especially for Portugal which has one of the largest proportion of elderly in the world [29]. As for the latter, most of the clusters were located in the interior of the countries, while the majority of the Brazilian and Portuguese populations live near the coastal regions. In Portugal, the existing FHUs are also concentrated along this region [21]. The reduced geographical proximity between primary health centers and patients can help explain the inequality between rural and urban areas. Not having a close provider of health services can be considered a barrier to access, because people can postpone seeking help until the condition requires hospitalization [52]. The remoteness of such areas can also be an obstacle to attracting and retaining health professionals [53]. For Portugal, it is important to note that the city of Lisbon (the most populated city in the country, with the fourth highest population density) presented high rates of both types of ACSC hospitalizations and was a high risk cluster on its own. The causes and possible associations of this finding should be studied further.

In Brazil, critical areas for acute conditions had a lower proportion of people living in rural areas. Previous studies have pointed out that the highest percentage of families registered at FHUs was in the rural areas of the country [54], and that accessibility and consolidation of PHC is a challenge in large urban centers [23]. Both Brazil and Portugal have FHUs coverage differential across their territories; therefore, the implementation and development of the PHC reforms were not uniform across each country. Results indicate that the PHC reforms, with similar organizational characteristics in different contexts, did not produce similar results between or within countries.

As for the stratification of ACSC between acute and chronic, the Spearman correlation between rates and the chi-square for the municipalities which belong or do not belong to clusters indicated a significant level of agreement between both categories. Nonetheless, the Mann-Whitney test indicated that the mean values of the ecological variables had contrasting differences between both categories for both Brazil and Portugal. Mostly, studies on hospitalizations for ACSC use this indicator as an aggregate of all the conditions deemed avoidable [17,55]. Results indicate that, although the identification of critical areas may be done using ACSC as an aggregated indicator, it is important to analyze the characteristics of these areas more deeply and separately when designing interventions, because the heterogeneity of mean values of the ecological variables could indicate that factors associated with each category of ACSC can be different. The findings of this study suggest the importance of using hospitalizations for ACSC to assess performance on a national level, while taking further actions to reduce them locally, given the context of each smaller region.

### Strengths and limitations

This study used large national databases covering all hospitalizations registered in public hospitals in Brazil and Portugal in 2015, as well as ecological data on different dimensions that can be associated with avoidable hospitalizations. A further strength of the study is represented by the well validated spatial scan approach used, which allows for local health authorities of both countries to identify critical regions to focus on, with important implications for health policy. This methodology can be expanded to other contexts as necessary. The comparison of hospitalizations for ACSC between Brazil and Portugal is a valuable opportunity to analyze variations between two settings with similar PHC organizations and important differences in country areas, demographics, epidemiologic characteristics, and levels of economic development.

One important limitation of this study is that, because of its ecological approach, it is not possible to establish causal relationships between variables. Nonetheless, this approach seems appropriate to analyze ACSC hospitalizations, because some studies recommend that this analysis should be performed at a group level [18,56]. We did not standardize the rates of ACSC hospitalizations, so the composition of populations had impact on results. Although to standardize rates is common practice to compare distinct contexts, we wanted to identify what are the real geographic areas that should receive more detailed attention. We wanted to identify these critical areas in real populations and analyze if they are related to similar characteristics, including ageing. For example, if prevalence of elderly was the only different characteristic between cluster and no cluster areas, it would mean that it was mostly important to improve older people health care. Our study showed that this is not the case. In addition, the ecologic variables were not standardized either.

The use of routinely collected administrative data is another limitation of this study, because the validity of diagnosis can vary according to ICD coding, across diseases, hospitals, and countries. Furthermore, the analysis performed only covers part of the complicated framework of factors associated with ACSC, because other important unobserved variables were not considered in this study.

## Conclusions

Brazil and Portugal presented substantial differences in rates of hospitalization for ACSC, geographic patterns, and characteristics of critical areas. They also presented expressive regional differences with regard to rates of hospitalization for ACSC, indicating that there is room to improve by reducing such events in both countries. The findings of this study show that different areas had different interactions between PHC supply and socioeconomic characteristics for both acute and chronic ACSC; thus, possible actions to reduce avoidable hospitalizations should be defined at a local level.
